# Clinical Utility of the PHQ‐2 and GAD‐2 in Pulmonary Hypertension

**DOI:** 10.1002/pul2.70299

**Published:** 2026-04-16

**Authors:** Gregg H. Rawlings, Jemma L. Green, Alexander M. K. Rothman, Abbie S. L. Barrell‐Stark, Andrew R. Thompson, Iain Armstrong

**Affiliations:** ^1^ Clinical and Applied Psychology Unit University of Sheffield Sheffield UK; ^2^ School of Psychology University of Sheffield Sheffield UK; ^3^ Division of Clinical Medicine, The University of Sheffield, and Sheffield Pulmonary Vascular Disease Unit, Royal Hallamshire Hospital Sheffield Teaching Hospitals NHS Foundation Trust Sheffield UK; ^4^ South Wales Clinical Psychology Training Programme Cardiff and Vale University Health Board and Cardiff University Cardiff UK

## Abstract

Integrating routine mental health support in pulmonary hypertension care is not just a matter of efficiency but also of recognition, equity, and epistemic justice. We provide evidence supporting the Generalised Anxiety Disorder‐2 questionnaire and the Patient Health Questionnaire‐2 as practical, psychometrically sound, and low‐burden entry points for screening anxiety and depression.

## 1 Introduction

Anxiety and depressive disorders are common in adults living with pulmonary hypertension (PH). A large scale meta‐analysis of more than 2000 patients with PH found pooled prevalence rates of 37% and 28%, respectively [1]. The conditions have been associated with reduced health‐related quality of life in PH and increased symptom burden and functioning [2]. While anxiety and depression are not always significantly related to severity of PH symptoms [1], they have been suggested to be linked with factors that worsen the condition [3, 4, 5].

There are only a few trials to date testing treatments specifically targeting anxiety or depression in PH [6, 7]; however, the inclusion of anxiety or depression specific patient‐reported outcome measures as secondary outcomes across trials is common [8]. Measures of anxiety and depression can be administered as part of care plans, and there is growing support for their routine integration in PH clinical practice [9, 10, 11].

Diagnostic testing is a corner stone of evidence‐based practice [12]. Indeed, the Generalised Anxiety Disorder‐7 Questionnaire (GAD‐7) [13] and Patient Health Questionnaire‐9 (PHQ‐9) [14] are two of the most administered measures to screen for anxiety and depression in PH [1]. Responders are asked about relevant symptoms over the last 2 weeks, reflecting the diagnostic criteria for generalised anxiety and depression reported in the Diagnostic and Statistical Manual of Mental Disorders. The measures are readability accessible, free to use, easy to interpret, well normed and supported by strong psychometric properties across clinical populations. A score of 8 or more on the GAD‐7 has a sensitivity of 89% and specificity of 82% for generalised anxiety disorder [13], and a score of 10 or more on the PHQ‐9 has a sensitivity of 88% and a specificity of 88% for major depression in the general population [14].

**Figure 1:**
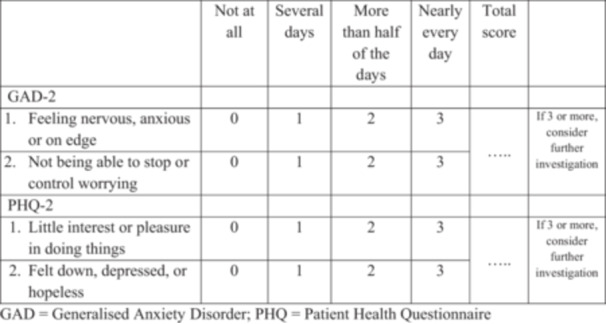
GAD‐2 and PHQ‐2 screening tool [15, 16, 17]. *Over the past 2 weeks, how often have you been bothered by any of the following problems?* GAD = Generalised Anxiety Disorder, PHQ = Patient Health Questionnaire.

While the measures are already relatively brief ‐ comprising of 7 and 9 items ‐ briefer version have been proposed named the GAD‐2 [15] and PHQ‐2 [16] (Figure 1). These are comprised of the first two items from each of the fuller version. A score of 3 or more on either measure is used a clinical cut off. These measures have been preferred in some PH research [18] and may be a quick, acceptable, and accurate screening tool, in the first instance, as they could be less burdensome on patients to complete and clinicians to administer. However, there may be a trade‐off between ease of use and accuracy, and therefore prior to their widespread use in PH, the two measures need to be psychometrically tested in this clinical group. This research letter compares the GAD‐7 and PHQ‐9 with their shorter versions using pre‐existing datasets, to assess whether the briefer scales perform comparably in PH.

## 2 Methods

Participants were recruited internationally through PH associations; therefore, the data reflect a community‐based sample. They responded to a series of psychological‐based research studies in PH that measured anxiety or depression using the GAD‐7 or PHQ‐9 – many of these studies have since been published. As such, it is likely that some responses came from the same individual. Therefore, we have refrained from reporting participant characteristics, as the values are unlikely to be reliable. Participants completed the measures online. All patients were eligible regardless of type of PH or functional class. Ethical approval was obtained for each study and participants provided consent for their data to be used for future research. Here we assess reliability, convergent validity, criterion validity, agreement, and construct validity. Statistical analyses were run on SPSS version 29 and R‐studio. ChatGTP was used to aid with analyses; outputs were edited as needed by authors who take full responsibility for the content of the publication.

## 3 Results

### 3.1 GAD

In total, 303 responses to the GAD‐7 were analysed. Average score on the GAD‐7 was 8.33 (SD = 6.12) and 2.53 (SD = 1.99) on the GAD‐2. Internal consistency of the GAD‐7 was excellent, whereas it was good for the GAD‐2 (0.93 and 0.89 respectively). Scores on the two measures were significantly and strongly correlated at 0.94, *p* < 0.001. A confirmatory factor analysis (CFA) was conducted to evaluate the performance of the two items that make up the GAD‐2, assuming the one‐factor structure of the GAD‐7. Model of fit indices were mixed: *χ*² [14] = 139.573, *p* = < 0.0001; CFI = 0.929; TLI = 0.893; RMSEA = 0.172; SRMR = 0.048. All seven items loaded moderately to very strongly on the latent factor (standardised loadings range = 0.642–0.927), with the two items from the GAD‐2 both demonstrating very strong loadings, 0.866 and 0.927 respectively.

Using the cut off on the GAD‐7 as a reference, a receiver operator characteristic (ROC) curve confirmed a score of 3 (2.5 or above) on the GAD‐2 was the most useful, with a Yoden's index value of 0.848 and a sensitivity of 0.866 and specificity of 0.981. The area under the curve was significant (0.968, *p* < 0.001). Agreement between below or above the cut off classification using the GAD‐7 and GAD‐2 were examined; 123 (40.6%) participants scored above the cut off on both measures, 158 (52.1%) scored below the cut off on both measures and 22 (7.3%) were misclassified. Overall agreement (92.7%) between the two measures on classification indicated a strong (Kappa level= 0.853, *p* < 0.001). The GAD‐2 demonstrated a sensitivity of 86.6%, specificity of 98.1%, positive predictive value of 97.6%, and negative predictive value of 89.2%.

### 3.2 PHQ

357 responses to the PHQ‐9 were analysed. Average score on the PHQ‐9 was 9.75 (SD = 6.43) and 2.15 (SD = 1.83) on the PHQ‐2. Internal consistency was good for both measures (0.886 and 0.841 respectively). Scores were significantly strongly correlated at 0.87, *p* < 0.001. A CFA was conducted on the PHQ‐9. Metrics were again mixed: χ²[27] = 179.934, *p* = < 0.001; CFI = 0.899; TLI = 0.866; RMSEA = 0.126; SRMR = 0.057. All items loaded moderately to very strongly on the latent depression factor (standardised loadings range = 0.505–0.871) – except for item 9 asking about suicide ideation (0.294). The PHQ‐2 items demonstrated the high loadings (0.782 and 0.819).

Compared to the PHQ‐9, a ROC curve confirmed a score of 3 (2.5 or more) on the PHQ‐2 provided the best result with a Yoden's index value of 0.641, a sensitivity of 0.683 and specificity of 0.959. The area under the curve was significant (0.901, *p* < 0.001). Examination of agreement between below or above the cut off classification using the PHQ‐9 and PHQ‐2 revealed, 112 (31.4%) participants scored above the cut off on both measures, 185 (51.8%) scored below the cut off on both measures and 60 (16.8%) scored differently. Overall agreement (83.19%) between the two measures on classification indicated a moderate agreement (Kappa level = 0.655, *p* < 0.001). The PHQ‐2 demonstrated a sensitivity of 93%, specificity of 78.1%, positive predictive value of 68.3%, and negative predictive value of 95.8%.

## 4 Discussion

This analysis confirms the GAD‐2 and PHQ‐2 are psychometrically sound tools for screening anxiety and depression in people with PH. Both measures demonstrated acceptable internal consistency and high correlations with their full‐length counterparts, supporting their use in clinical and research settings. However, the findings also reveal important distinctions in diagnostic accuracy and interpretive nuance.

The GAD‐2 performed exceptionally well, showing strong agreement with the GAD‐7 and high sensitivity and specificity. This suggests it can reliably identify individuals experiencing anxiety with minimal burden ‐ a critical consideration in PH care, where cognitive load and symptom fatigue are common. In contrast, the PHQ‐2, while useful, showed lower agreement and a higher misclassification rate. This underscores the value of the full PHQ‐9 in capturing the complexity of depressive symptoms.

The results also raise questions about the underlying structure of these measures in PH populations. Mixed model fit indices suggest that a single‐factor model may not fully capture the symptom profile, particularly for depression. This invites further exploration including whether cognitive‐emotional and somatic dimensions should be considered separately in PH‐specific adaptations.

It is a limitation that the GAD‐7 and PHQ‐9 were used as a reference for determining cut off scores, rather than a more comprehensive assessment to evaluate whether an individual would meet criteria for anxiety or depression, such as the Clinical Interview Schedule‐Revised. It is possible a cut off of 8 and 10 is not appropriate for this clinical group, as it may not consider the impact of cardiovascular symptoms on scores. The cross‐sectional and retrospective nature of the dataset limits what additional questions we could have asked, such as whether screening accuracy of depression and anxiety is related to PH factors. Finally, as the dataset could have included answers from the same patient, it violates the assumption of independence of observations possibly increasing the possibility of type 1 errors.

To conclude, this study highlights a broader issue; despite the availability of validated tools, many individuals with PH and clinical levels of anxiety or depression remain untreated. The GAD‐2 and PHQ‐2 offer a practical, low‐burden entry point for routine psychological screening, but their use must be embedded within a care pathway that recognises distress as clinically significant and worthy of intervention. Such tools help give voice to the emotional and cognitive burdens that often go unseen. In doing so, they enable the lived experiences of patients to be heard with greater clarity, legitimacy, and clinical relevance.

## Author Contributions

Gregg H. Rawlings developed the concept of the study and was involved in data collection and analysis, and writing the report for publication. Jemma L. Green was involved in data collection and write up. Alexander M. K. Rothman developed the concept of the study and was involved in writing the report for publication. ASLB was involved in data collection and write up. Alexander M. K. RothmanT was involved in data collection and write up. Iain Armstrong was involved in data collection and write up. All authors approved the final version for publication.

## Funding

The authors have nothing to report.

## Ethics Statement

Initial studies obtained ethical approval from The University of Sheffield Psychology Ethics Committee (035318, 034442 and 067112), Schools of Business, Law and Social Sciences Research Ethics Committee at Nottingham Trent University (2021/417) and Cardiff University Psychology Ethics Committee (EC.22.12.13.6673R2A).

## Consent

The authors have nothing to report.

## Conflicts of Interest

The authors declare no conflicts of interest.
